# Association between *EGFR* Gene Mutation and Antioxidant Gene Polymorphism of Non-Small-Cell Lung Cancer

**DOI:** 10.3390/diagnostics10090692

**Published:** 2020-09-14

**Authors:** Ching-Hsiung Lin, Po-Jen Yang, Sheng-Hao Lin, Kun-Tu Yeh, Thomas Chang-Yao Tsao, Yu-En Chen, Shu-Hui Lin, Shun-Fa Yang

**Affiliations:** 1 Division of Chest Medicine, Department of Internal Medicine, Changhua Christian Hospital, Changhua 500, Taiwan; 47822@cch.org.tw (C.-H.L.); 112364@cch.org.tw (S.-H.L.); 2 Institute of Genomics and Bioinformatics, National Chung Hsing University, Taichung 402, Taiwan; 3 Department of Recreation and Holistic Wellness, MingDao University, Changhua 500, Taiwan; 4 School of Medicine, Chung Shan Medical University, Taichung 402, Taiwan; cshy1030@csh.org.tw (P.-J.Y.); 10159@cch.org.tw (K.-T.Y.); tcyt@csmu.edu.tw (T.C.-Y.T.); 5 Department of Family and Community Medicine, Chung Shan Medical University Hospital, Taichung 402, Taiwan; 6 Department of Surgical Pathology, Changhua Christian Hospital, Changhua 500, Taiwan; 182242@cch.org.tw; 7 Division of Chest, Department of Internal Medicine, Chung Shan Medical University Hospital, Taichung 402, Taiwan; 8 Department of Medical Laboratory Science and Biotechnology, Central Taiwan University of Science and Technology, Taichung 402, Taiwan; 9 Institute of Medicine, Chung Shan Medical University, Taichung 402, Taiwan; 10 Department of Medical Research, Chung Shan Medical University Hospital, Taichung 402, Taiwan

**Keywords:** adenocarcinoma, *EGFR* mutation, antioxidant gene polymorphisms

## Abstract

*EGFR* mutation status is considered as an important predictor of therapeutic responsiveness in non-small-cell lung carcinoma patients. Recent evidence suggests that antioxidant gene polymorphisms are potential predictors of lung cancer risk. Thus, stratification of *EGFR* mutation-related phenotypes by antioxidant gene polymorphism status can be an effective approach in terms of improving the prognosis of lung cancer patients. The present study was designed to evaluate the distribution frequency of antioxidant gene polymorphisms in lung adenocarcinoma, as well as its association with hotspot *EGFR* mutations. The study findings revealed that a statistically significant association exists between *EGFR* L858R mutation and AG + GG genotypes of *SOD* rs4880 polymorphism. Furthermore, the subgroup analysis data revealed that compared to AA genotype of *SOD* rs4880, AG + GG genotypes were significantly associated with advanced cancer stage and distant metastasis. Taken together, these findings can be utilized clinically to predict cancer aggressiveness, metastatic, potential and therapeutic responsiveness of lung cancer patients.

## 1. Introduction

Lung cancer is the leading cause of death worldwide. It is a highly invasive and metastatic cancer with very poor prognosis. The death rate associated with lung cancer is higher than that observed in colon, breast and pancreatic carcinomas, and the 5-year survival rate of lung cancer is lower than 18% [1]. Among all lung cancer types, small-cell lung carcinoma and non-small-cell lung carcinoma (squamous-cell carcinoma, adenocarcinoma, and large-cell carcinoma) account for 15% and 85%, respectively [2].

Epidermal growth factor receptor (EGFR) is a transmembrane protein with a tyrosine kinase domain in the intracellular portion. It plays important roles in regulating various physiological functions, including cell proliferation and migration [3]. Activating mutations in *EGFR* gene are observed frequently in non-small-cell lung carcinoma patients [4], and *EGFR* gene polymorphisms are associated with the risk of lung cancer [5]. It is well-documented that the most common *EGFR* mutations including exon 19 deletions and L858R mutations strongly predict the sensitivity of lung cancer patients to tyrosine kinase inhibitor treatments [6]. Moreover, studies conducted on erlotinib-treated lung cancer patients with *EGFR* mutations have shown that a specific polymorphism (181946C > T) in *EGFR* gene is associated with long-term progression-free and overall survival [7].

Reactive oxygen species (ROS) are produced as a byproduct of cellular respiration and act as a second messenger in various signaling pathways. However, excessive production of ROS can lead to initiation of a series of cellular reactions that ultimately cause oxidative stress and inflammation, which are two hallmarks of cancer onset and progression [8]. To counterbalance the burden of increased ROS, there are a wide variety of nonenzymatic antioxidants, such as glutathione, carotenoids, flavonoids and vitamins A, C and E, as well as enzymatic antioxidants, such as superoxide dismutase (SOD), catalase (CAT), glutathione peroxidase (GPX) and thioredoxin (TRX) [8].

A number of studies have been conducted to evaluate the effect of antioxidant system on cancer progression and recurrence [9]. In this context, one interesting study revealed that production of glutathione is a prerequisite for cancer onset, but not for cancer progression [10]. Moreover, the study has pointed out that inhibition of both glutathione and TRX signaling pathways can induce cancer cell apoptosis. The activity of antioxidant system is particularly important in lung carcinomas as lung tissue is highly susceptible to oxidative stress-induced damage [11]. One recent study has demonstrated that the activities of different SOD isotypes and catalase are low in lung cancer cells than adjacent normal cells; however, the activities of GPX, glutathione reductase, and glutathione S-transferase are higher in lung cancer cells [12].

Genetic polymorphisms of cellular antioxidants are known to play a significant role in the pathogenesis of various oxidative stress- and inflammation-related diseases, such as cancer [13,14,15,16]. Recently, a meta-analysis study has stated that catalase C262 T polymorphism is associated with an increased risk of prostate cancer [17]. Similarly, polymorphisms in *SOD* gene is associated with the onset of different cancer types, including lung and colorectal cancers [18]. In case of lung cancer, one study has shown that glutathione S-transferase T1 gene polymorphism is associated with the lung cancer risk [19]. Similarly, manganese *SOD* (*MnSOD*) gene polymorphisms (Ala16Val) together with the smoking status are known to be associated with an increased risk of lung cancer [20]. In *EGFR*-mutated non-small-cell lung cancer patients treated with tyrosine kinase inhibitors, genetic polymorphisms of glutathione S-transferase P1, myeloperoxidase and biliverdin reductase A have been shown to be associated with a reduced overall survival [21]. Interestingly, it has been found that EGFR-overexpressing breast cancer cells can develop resistance against tyrosine kinase inhibitors due to an increased cellular ROS level, indicating that antioxidant-mediated elimination of excessive ROS can be a potential strategy to treat patients with *EGFR*-mutated carcinomas [22]. Previous studies showed association between SNPs and antioxidant genes in various cancer types, including breast, prostate, colon and lung cancer [23,24,25]. These SNPs affect protein or mRNA expression in human cancers. Based on these study, in this present study we aimed to explore the correlation between SNPs and *EGFR* mutation in NSCLC patients.

## 2. Materials and Methods

### 2.1. Patients

A total of 314 patients with lung adenocarcinoma (age range: 30–70 years) were recruited for the present study. Of all patients, 117 had lung adenocarcinoma with wild-type *EGFR* and 197 had *EGFR*-mutated lung adenocarcinoma. Demographic characteristics and medical information of the patients, including gender, smoking status, AJCC clinical staging, tumor ‘T’ classification, lymph node status, distant metastasis, and tumor differentiation, were obtained from their medical records. For *EGFR* gene sequencing, paraffin-embedded cancer tissues were collected; in addition, whole blood samples were collected from the participants and kept in EDTA-containing sterile tubes for genotyping. This study was approved by the Institutional Review Board (IRB) of Changhua Christian Hospital at Changhua (IRB No. 140205, date of approval 9 March 2014) and Cheng-Ching General Hospital (No. HP120009, 22 September 2012).

### 2.2. EGFR Mutation Testing

Paraffin-embedded tumor tissues were used to extract DNA using cobas^®^ DNA Sample Preparation Kit (Roche, Indianapolis, IN, USA). Real-time quantitative PCR (high resolution melting analysis) analyzed with cobas^®^ Z480 (Roche, Basel, Switzerland). The cobas *EGFR* Mutation Test v2 can identify 42 mutations in exons 18, 19, 20 and 21 of the *EGFR* gene, including G719X, ex19del, S768I, T790M, exon 20 insertions, L858R and L861Q.

### 2.3. Genotyping

The whole blood samples were used to extract the genomic DNA using QIAamp DNA blood mini kits (Qiagen, Valencia, CA, USA) following the manufacturer’s instructions. The polymorphisms of different antioxidant genes, *SOD* rs5746136 (assay IDs: C__29322854_10); *SOD* rs4880 (assay IDs: C___8709053_10); *CAT* rs769218 (assay IDs: C___3102900_10); *OGG1* rs1052133 (assay IDs: C___3095552_1_); and *TXN2* rs4821494 (assay IDs: C___2457432_10) were determined by real-time PCR genotyping using the ABI StepOne™ real-time PCR system (Applied Biosystems, Foster City, CA, USA). The results were analyzed using the StepOnePlus™ Software v2.3 (Applied Biosystems).

### 2.4. Statistical Analysis

The distributions of patient demographic features and genotype frequencies between wild-type and *EGFR*-mutated lung adenocarcinomas, as well as the clinicopathological features of *EGFR*-mutated lung adenocarcinoma patients in polymorphic genotypes of *SOD* rs4880 were analyzed by χ^2^-test. After controlling for other covariables, the odds ratio and 95% CIs of the association between the genotype frequencies and *EGFR* mutation risk and the clinicopathological features were calculated using multiple logistic regression models. A *p* value of <0.05 was considered statistically significant. All statistical analyses were carried out using SAS statistical software (Version 9.1, 2005; SAS Institute, Cary, NC, USA).

## 3. Results

### 3.1. Demographic and Clinical Characteristics of Participants

A total of 314 lung adenocarcinoma patients were enrolled for the study. Table 1 shows the demographic and clinical details of the participant. Of all participants, 117 had lung adenocarcinoma with wild-type *EGFR* (the WT group) and 197 had *EGFR*-mutated lung adenocarcinoma (the *EGFR*-mutated group). The average ages of the participants in the WT and *EGFR*-mutated groups were 65.7 and 65.1, respectively. There were statistically significant differences between the WT and *EGFR*-mutated groups in regard to gender, smoking status, tumor ‘T’ classification and tumor differentiation. Than the WT group, the *EGFR*-mutated group had higher numbers of female (126 vs. 48) and non-smoker (158 vs. 57) participants. Moreover, the numbers of participants with well- (26 vs. 7) and moderately differentiated (158 vs. 89) tumors were higher in the *EGFR*-mutated group.

### 3.2. Distribution of Antioxidant Gene Polymorphisms of Participants and Its Association with EGFR Mutation

Table 2 shows the distribution frequency of antioxidant gene polymorphisms (*SOD* rs5746136 and *SOD* rs4880; *CAT* rs769218; *OGG1* rs1052133; and *TXN2* rs4821494) of lung adenocarcinoma patients. The alleles with the highest distribution frequency for *SOD* rs5746136 and *SOD* rs4880 in study participants were homozygous C/C and homozygous A/A for both the WT and *EGFR*-mutated groups, respectively. For *CAT* rs769218; *OGG1* rs1052133; and *TXN2* rs4821494, the alleles with the highest distribution frequency among participants were homozygous G/G, homozygous C/C and homozygous G/G, respectively, for both the WT and *EGFR*-mutated groups. For all antioxidant gene polymorphisms, there was no statistically significant association between different genotypes and *EGFR* mutation status in lung adenocarcinoma patients.

### 3.3. Association between Antioxidant Gene Polymorphisms and EGFR Hotspot Mutations in Lung Adenocarcinoma

Table 3 represents the association between antioxidant gene polymorphisms (*SOD* rs5746136 and *SOD* rs4880; *CAT* rs769218; *OGG1* rs1052133; and *TXN2* rs4821494) and *EGFR* hotspot mutations in study participants. In the present study, two hotspot *EGFR* mutations including L858R and exon 19 in-frame deletions were evaluated. A significant association between L858R mutation and AG + GG genotypes of *SOD* rs4880 polymorphism was observed (AOR = 1.90; 95% CI = 1.01–3.58; *p* = 0.047). However, there was no statistically significant association between *SOD* rs4880 genotypes and exon 19 in-frame deletions. For *SOD* rs5746136, *CAT* rs769218, *OGG1* rs1052133 and *TXN2* rs4821494 polymorphisms, no statistically significant association was observed between different genotypes and *EGFR* hotspot mutations.

### 3.4. Subgroup Analysis of EGFR Mutations Based on Polymorphic Genotypes of SOD rs4880

To evaluate the correlation between clinicopathological features of *EGFR* hotspot mutations and *SOD* rs4880, a subgroup analysis of all lung adenocarcinoma cases as well as *EGFR* L858R and Exon 19 deletion mutations were performed (Table 4). The subgroup analysis data revealed that compared to AA genotype, AG + GG genotypes of *SOD* rs4880 were associated with more aggressive lung adenocarcinoma phenotypes in terms of cancer staging, tumor size, lymph node status, distant metastasis and tumor differentiation. Moreover, a statistically significant correlation was observed between AG + GG genotypes and higher cancer staging irrespective of the *EGFR* mutation status. When considering all lung adenocarcinoma patients (WT and *EGFR*-mutated), AG + GG genotypes were found to correlate significantly with distant metastasis; however, similar statistically significant correlation was not observed for *EGFR*-mutated tumors.

### 3.5. Subgroup Analysis of EGFR Mutations in Non-Smoking Patients Based on Polymorphic Genotypes of SOD rs4880

Finally, we evaluated the correlation between clinicopathological features in non-smoking NSCLC patients with *EGFR* mutations and *SOD* rs4880 (Table 5). The subgroup analysis data revealed that compared to AA genotype, AG + GG genotypes of *SOD* rs4880 were associated with more aggressive lung adenocarcinoma phenotypes in terms of cancer staging (III + IV). Moreover, a statistically significant correlation was observed between AG + GG genotypes and higher cancer staging with non-smoking status (AOR = 3.15; 95% CI = 1.28–7.72; *p* = 0.012). Especially highly significant correlation was observed in female non-smoking populations (AOR = 3.55; 95% CI = 1.25–10.13; *p* = 0.047).

## 4. Discussion

The present study was designed to evaluate the association between antioxidant gene polymorphisms (*SOD* rs5746136 and *SOD* rs4880; *CAT* rs769218; *OGG1* rs1052133; and *TXN2* rs4821494) and *EGFR*-mutated lung adenocarcinoma. To the best of our knowledge, this is the first study of its kind, and the study findings may help identify more appropriate treatment approaches for better cancer management.

Our demographical and clinical data indicated that the number of female and non-smoker participants as well as participants with well- and moderately differentiated tumors was significantly higher in the *EGFR*-mutated group compared to that in the WT group (Table 1). While studying the distribution frequency of antioxidant gene polymorphisms in patients with WT- or *EGFR*-mutated lung adenocarcinoma, we found no statistically significant association between different polymorphic genotypes and *EGFR* mutation status (Table 2). The analysis performed to investigate the relationship between antioxidant gene polymorphisms with *EGFR* hotspot mutations (L858R and Exon 19 deletion mutations) revealed that a statistically significant association exists between L858R mutation and AG + GG genotypes of *SOD* rs4880 polymorphism (Table 3). Furthermore, our subgroup analysis data revealed that AG + GG genotypes of *SOD* rs4880 were associated with more aggressive lung adenocarcinoma phenotypes compared to AA genotype of *SOD* rs4880. In particular, AG + GG genotypes were found to be correlated significantly with higher tumor stage and tumors with distant metastasis (Table 4). Additionally, we analyzed for clinical correlation in non-smoking NSCLC patients with *EGFR* mutations and *SOD* rs4880 (Table 5). Increased risk was observed in females; non-smokers; and with *EGFR* mutation.

The analysis of antioxidant gene polymorphisms in different cancer types has gained interest because of the significant impact of oxidant/antioxidant balance in the onset and progression of cancer [26,27]. There is a growing-pool of evidence suggesting that antioxidant gene polymorphisms are important predictors of cancer risks [18,19,20]. These studies have indicated that the distribution frequency of different polymorphic genotypes varies between different cancer subtypes, indicating the potential value of antioxidant gene polymorphisms as cancer biomarkers [28]. Moreover, genetic polymorphism-driven changes in the activity of cellular antioxidants can alter the therapeutic responsiveness of cancer patients through increased oxidative stress; thus, genetic polymorphism as a biomarker can be effective in identifying patient-specific therapeutic interventions and augmenting the responsiveness of personalized medicines [29]. The current findings showed that genetic variants in oxidative stress related genes may modify prognosis in EGFR TKIs-treated NSCLC patients. Cellular redox state is associated with the efficacy of EGFR TKIs treatment in NSCLC patients with activating *EGFR* mutations [21]. SOD2 overexpression was related to metastatic phenotype in cancers [30,31,32]. Several researchers have hypothesized that SOD2 overexpression promotes metastasis by increasing the steady-state concentration of H2O2 [33,34]. In our findings, AG + GG genotypes of *SOD* rs4880 were associated with more aggressive lung adenocarcinoma phenotypes compared to AA genotype of *SOD* rs4880 in advance NSCLC. Furthermore, we speculate that, AG + GG genotypes of *SOD* rs4880 increase SOD and promote NSCLC progression, especially in non-smoking female. Unfortunately, in this study, we do not have survival data to further validate the role of clinicopathological parameters in SNP by the Kaplan–Meier method or multivariate analysis.

Of all antioxidant genes, *SOD* genetic polymorphism is well-documented in the literature. SOD being the major first-line antioxidant neutralizes highly reactive superoxide free radicals into less reactive hydrogen peroxide [20]. In the present study, the in-depth analysis of the relationship between polymorphic genotypes of *SOD* rs4880 and clinic-pathologic features of *EGFR*-mutated lung adenocarcinoma is particularly important, because *SOD* polymorphism-mediated stratification of *EGFR* mutation-related phenotypes, which are considered to be a major predictor of therapeutic responsiveness, can be more advantageous and effective in predicting lung cancer risk as well as therapeutic responsiveness.

## 5. Conclusions

The present study findings revealed that *SOD* rs4880 polymorphism is significantly associated with a specific *EGFR* hotspot mutation, L858R. Moreover, AG + GG polymorphic genotypes of *SOD* rs4880 are significant correlated with advancer cancer stage and distant metastasis in *EGFR*-mutated lung adenocarcinoma patients. These findings can be utilized clinically to predict cancer aggressiveness, metastatic potential and therapeutic responsiveness.

## Figures and Tables

**Table 1:** Distributions of demographical characteristics in 314 patients with lung adenocarcinoma by *EGFR* mutation status.

Variable	Wild-Type	*EGFR* Mutation	Total	*p* Value
	*n* = 117 (%)	*n* = 197 (%)	*n* = 314	
Age				
<30	1 (0.9)	1 (0.5)	2	0.673
30–39	2 (1.7)	2 (1.0)	4	
40–49	10 (8.5)	19 (9.6)	29	
50–59	23 (19.7)	54 (27.4)	77	
60–69	29 (24.8)	40 (20.3)	69	
≥70	52 (44.4)	81 (47.1)	133	
Mean ± SD	65.7±12.7	65.1±13.2		0.512
Cigarette smoking				
Non-smoker	57 (48.7)	158 (80.0)	215	<0.001 *
Smoker	60 (51.3)	39 (19.8)	99	
Gender				
Female	48 (41.0)	126 (64.0)	174	<0.001 *
Male	69 (59.0)	71 (36.0)	140	
Tumor “T” classification				
T1	21 (17.9)	43 (21.8)	64	0.001 *
T2	42 (35.9)	85 (43.1)	127	
T3	27 (23.1)	14 (7.1)	41	
T4	27 (23.1)	55 (27.9)	82	
Lymph node status				
Negative	34 (29.1)	67 (34.0)	101	0.364
Positive	83 (70.9)	130 (66.0)	213	
Distant metastasis				
Negative	54 (46.2)	89 (45.2)	143	0.867
Positive	63 (53.8)	108 (54.8)	171	
Tumor AJCC staging				
I	23 (19.7)	45 (22.8)	68	0.619
II	7 (6.0)	8 (4.1)	15	
III	24 (20.5)	32 (16.2)	56	
IV	63 (53.8)	112 (56.9)	175	
Tumor differentiation				
Well	7 (6.0)	26 (13.2)	33	0.002 *
Moderate	89 (76.1)	158 (80.2)	247	
Poor	21 (17.9)	13 (6.6)	34	

SD is the abbreviation of standard Deviation. AJCC is the abbreviation of American Joint Committee on Cancer. The AJCC Cancer Staging Manual remains the gold standard reference for oncologists, surgeons, pathologists, radiologists, cancer registrars and medical professionals world-wide to ensure that all those caring for cancer patients are fully versed in the language of cancer staging. *: *p* value is less than 0.05.

**Table 2:** Distribution frequency of antioxidant gene polymorphism with lung adenocarcinoma and logistic regression of *EGFR* mutation association.

Variable	Wild-Type	*EGFR* Mutation	AOR	*p* Value
	*n* = 117 (%)	*n* = 197 (%)	95% CI	
*SOD* rs5746136				
CC	56 (47.9)	89 (45.2)	1.00	
CT	45 (38.5)	75 (38.1)	1.06 (0.63–1.76)	0.833
TT	16 (13.7)	33 (16.8)	1.40 (0.69–2.84)	0.347
CT + TT	61 (62.1)	108 (5438)	1.15 (0.72–1.83)	0.574
*SOD* rs4880				
AA	88 (75.2)	133 (67.5)	1.00	
AG	28 (23.9)	56 (28.4)	1.28 (0.75–2.20)	0.372
GG	1 (0.9)	8 (4.1)	4.96 (0.60–41.20)	0.139
AG + GG	29 (24.8)	64 (32.5)	1.41 (0.83–2.39)	0.204
*CAT* rs769218				
GG	61 (52.1)	99 (50.3)	1.00	
GA	35 (29.9)	44 (22.3)	0.78 (0.45–1.38)	0.391
AA	21 (17.9)	54 (27.4)	1.49 (0.81–2.74)	0.202
GA + AA	56 (47.9)	98 (49.7)	1.05 (0.66–1.68)	0.835
*OGG1* rs1052133				
CC	57 (48.7)	95 (48.2)	1.00	
CG	40 (34.2)	67 (34.0)	0.96 (0.57–1.62)	0.870
GG	20 (17.1)	35 (17.8)	0.89 (0.46–1.73)	0.729
CG + GG	60 (51.3)	102 (51.8)	0.93 (0.58–1.50)	0.777
*TXN2* rs4821494				
GG	55 (47.0)	107 (54.3)	1.00	
GT	54 (46.2)	73 (37.1)	0.62 (0.42–1.13)	0.143
TT	8 (6.8)	17 (8.6)	1.07 (0.42–2.70)	0.892
GT + TT	62 (53.0)	90 (45.7)	0.74 (0.46–1.19)	0.211

Adjusted odds ratios (AORs) with 95% confidence intervals (CIs) estimated by logistic models after controlling for age and gender.

**Table 3:** Associations between antioxidant gene polymorphism and epidermal growth factor receptor hotspot mutations in lung adenocarcinoma.

Variable	Wild-Type	Exon 19 in-Frame Deletion			L858R		
	*n* = 117 (%)	*n* = 96 (%)	AOR (95% CI)	*p* Value	*n* = 93 (%)	AOR (95% CI)	*p* Value
*SOD* rs5746136							
CC	56 (47.9)	45 (46.9)	1.00		42 (45.2)	1.00	
CT	45 (38.5)	34 (35.4)	0.90 (0.49–1.65)	0.731	39 (41.9)	1.29 (0.69–2.41)	0.433
TT	16 (13.7)	17 (17.7)	1.41 (0.63–3.16)	0.401	12 (12.9)	1.04 (0.42–2.56)	0.939
CT+TT	61 (62.1)	51 (53.1)	1.03 (0.59–1.79)	0.919	51 (54.8)	1.22 (0.68–2.19)	0.508
*SOD* rs4880							
AA	88 (75.2)	70 (72.9)	1.00		57 (61.3)	1.00	
AG	28 (23.9)	23 (24.0)	0.95 (0.50–1.83)	0.884	31 (33.3)	1.69 (0.88–3.25)	0.114
GG	1 (0.9)	3 (3.1)	3.68 (0.36–37.90)	0.273	5 (5.4)	8.68 (0.85–89.12)	0.069
AG + GG	29 (24.8)	26 (27.1)	1.04 (0.56–1.96)	0.894	36 (38.7)	1.90 (1.01–3.58)	0.047 *
*CAT* rs769218							
GG	61 (52.1)	50 (52.1)	1.00		43 (46.2)	1.00	
GA	35 (29.9)	22 (22.9)	0.80 (0.41–1.56)	0.519	22 (23.7)	0.90 (0.45–1.82)	0.772
AA	21 (17.9)	24 (25.0)	1.39 (0.68–2.83)	0.365	28 (30.1)	1.78 (0.86–3.69)	0.122
GA + AA	56 (47.9)	46 (47.9)	1.03 (0.59–1.79)	0.925	50 (53.8)	1.24 (0.69–2.22)	0.467
*OGG1* rs1052133							
CC	57 (48.7)	48 (50.0)	1.00		44 (47.3)	1.00	
CG	40 (34.2)	29 (30.2)	0.85 (0.45–1.61)	0.624	34 (36.6)	1.25 (0.65–2.38)	0.506
GG	20 (17.1)	19 (19.8)	0.98 (0.46–2.11)	0.965	15 (16.1)	0.88 (0.38–2.01)	0.757
CG + GG	60 (51.3)	48 (50.0)	0.90 (0.51–1.57)	0.707	49 (52.7)	1.11 (0.62–1.99)	0.723
*TXN2* rs4821494							
GG	55 (47.0)	54 (56.3)	1.00		48 (51.6)	1.00	
GT	54 (46.2)	37 (38.5)	0.69 (0.39–1.23)	0.206	33 (35.5)	0.65 (0.35–1.21)	0.176
TT	8 (6.8)	5 (5.2)	0.71 (0.21–2.36)	0.575	12 (12.9)	1.50 (0.53–4.24)	0.444
GT + TT	62 (53.0)	42 (43.7)	0.69 (0.40–1.21)	0.194	45 (48.4)	0.76 (0.42–1.37)	0.367

Adjusted odds ratios (AORs) with 95% confidence intervals (CIs) estimated by logistic regression models after controlling for age and gender. *: *p* value is less than 0.05.

**Table 4:** Clinicopathologic characteristics of lung adenocarcinoma patients with *EGFR* mutation, stratified by polymorphic genotypes of *SOD* rs4880.

	All Cases (*n* = 306)				L858R and Exon 19 Deletion (*n* = 189)			
Variable	AA (*n* = 215)	AG + GG (*n* = 91)	AOR (95% CI)	*p* Value	AA (*n* = 127)	AG + GG (*n* = 62)	AOR (95% CI)	*p* Value
Stage								
I + II	66 (30.7)	15 (16.5)	1.00		41 (32.3)	10 (16.1)	1.00	
III + IV	149 (69.3)	76 (83.6)	2.24 (1.20–4.22)	0.012 *	68 (67.7)	52 (83.9)	2.40 (1.10–5.22)	0.027 *
Tumor T status								
T1 + T2	134 (62.3)	53 (58.2)	1.00		84 (66.1)	40 (64.5)	1.00	
T3 + T4	81 (37.7)	38 (41.8)	1.18 (0.71–1.95)	0.523	43 (33.9)	22 (35.5)	1.02 (0.53–1.95)	0.951
Lymph node status								
Negative	76 (35.3)	23 (25.3)	1.00		48 (37.8)	17 (27.4)	1.00	
Positive	139 (64.7)	68 (74.7)	1.63 (0.94–2.84)	0.084	79 (62.2)	45 (72.6)	0.56 (0.80–3.30)	0.195
Distant metastasis								
Negative	106 (49.3)	33 (36.3)	1.00		63 (49.6)	22 (35.5)	1.00	
Positive	109 (50.7)	58 (63.7)	1.69 (1.01–2.80)	0.044 *	64 (50.4)	40 (64.5)	1.72 (0.92–3.25)	0.092
Tumor differentiation								
Well	24 (11.2)	7 (7.7)	1.00		19 (15.0)	5 (8.1)	1.00	
Moderate + poor	191 (88.8)	84 (92.3)	1.52 (0.63–3.69)	0.353	108 (85.0)	57 (91.9)	1.98 (0.70–5.60)	0.198

I + II is population of stage I and stage II, we compared early stage and advance stage. T1 + T2 is population of T1 and T2, this is only analysis tumor size and genetic variants association. AOR is the abbreviation of adjusted odds ratios, adjust gender and age. CI is the abbreviation of confidence interval. *: *p* value is less than 0.05.

**Table 5:** Clinicopathologic characteristics of lung adenocarcinoma patients with *EGFR* mutation in non-smoking population, stratified by polymorphic genotypes of *SOD* rs4880.

	*EGFR* Mutation in Non-Smoking Cases (*n* = 155)				*EGFR* Mutation in Female with Non-Smoking Cases (*n* = 121)			
Variable	AA (*n* = 204)	AG+GG (*n* = 51)	AOR (95% CI)	*p* Value	AA (*n* = 83)	AG+GG (*n* = 38)	AOR (95% CI)	*p* Value
Stage								
I+II	35 (33.7)	7 (13.7)	1.00		30 (36.1)	5 (13.2)	1.00	
III+IV	69 (66.3)	44 (86.3)	3.15 (1.28–7.72)	0.012 *	53 (63.9)	33 (86.8)	3.55 (1.25–10.13)	0.018 *
Tumor T status								
T1+T2	69 (66.3)	33 (64.7)	1.00		57 (68.7)	24 (63.2)	1.00	
T3+T4	35 (33.7)	18 (35.3)	1.06 (0.53–2.15)	0.865	26 (31.3)	14 (36.8)	1.19 (0.53–2.71)	0.671
Lymph node status								
Negative	41 (39.4)	14 (27.5)	1.00		35 (42.2)	11 (28.9)	1.00	
Positive	63 (60.6)	37 (72.5)	1.69 (0.81–3.52)	0.161	48 (57.8)	27 (71.1)	1.76 (0.77–4.03)	0.184
Distant metastasis								
Negative	52 (50.0)	18 (35.3)	1.00		45 (54.2)	13 (34.2)	1.00	
Positive	52 (50.0)	33 (64.7)	1.80 (0.90–3.62)	0.097	38 (45.8)	25 (65.8)	2.15 (0.96–4.82)	0.063
Tumor differentiation								
Well	15 (14.4)	4 (7.8)	1.00		13 (15.7)	2 (5.3)	1.00	
Moderate + poor	89 (85.6)	47 (92.4)	1.98 (0.62–6.30)	0.250	70 (84.3)	36 (94.7)	3.24 (0.69–15.20)	0.137

I + II is population of stage I and stage II, we compared early stage and advance stage. T1 + T2 is population of T1 and T2, this is only analysis tumor size and genetic variants association. AOR is the abbreviation of adjusted odds ratios, adjust gender and age. CI is the abbreviation of confidence interval. *: *p* value is less than 0.05.
